# Supplementary material for: Soil Microbes Mediate Productivity Differences Between Natural and Plantation Forests
Source: Plants (Basel). 2025 Dec 28;15(1):98. doi: 10.3390/plants15010098 (PMC12787416; doi:10.3390/plants15010098)
Supplement: Supplementary file 1 [file plants-15-00098-s001.zip › plants-4035602-supplementary.pdf]

Supplementary file

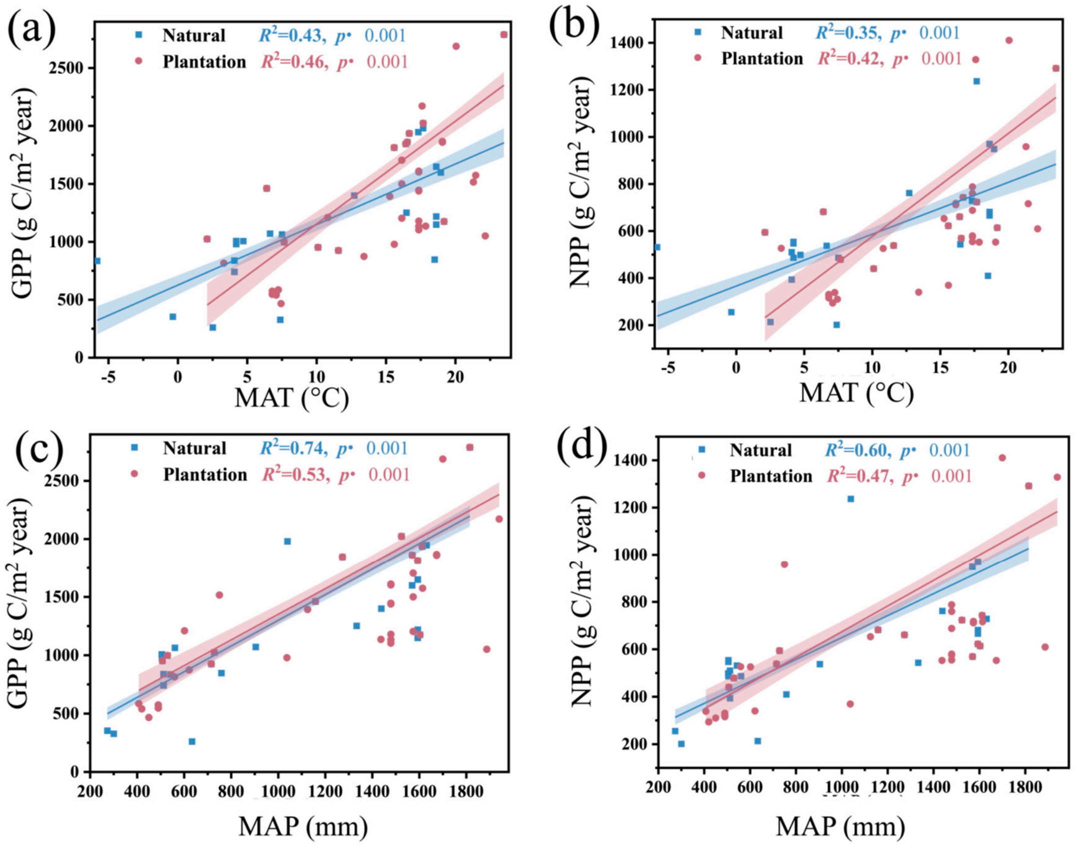

Figure. S1. Relationships between Climatic Factors (Annual Mean Temperature, Annual Precipitation) and Forest Productivity (Gross Primary Productivity (GPP), Net Primary Productivity (NPP)): (a) Annual Mean Temperature and GPP; (b) Annual Mean Temperature and NPP; (c) Annual Precipitation and GPP; (d) Linear Regression of Annual Precipitation and NPP. R^2 represents the goodness of fit, and the p-value indicates statistical significanc

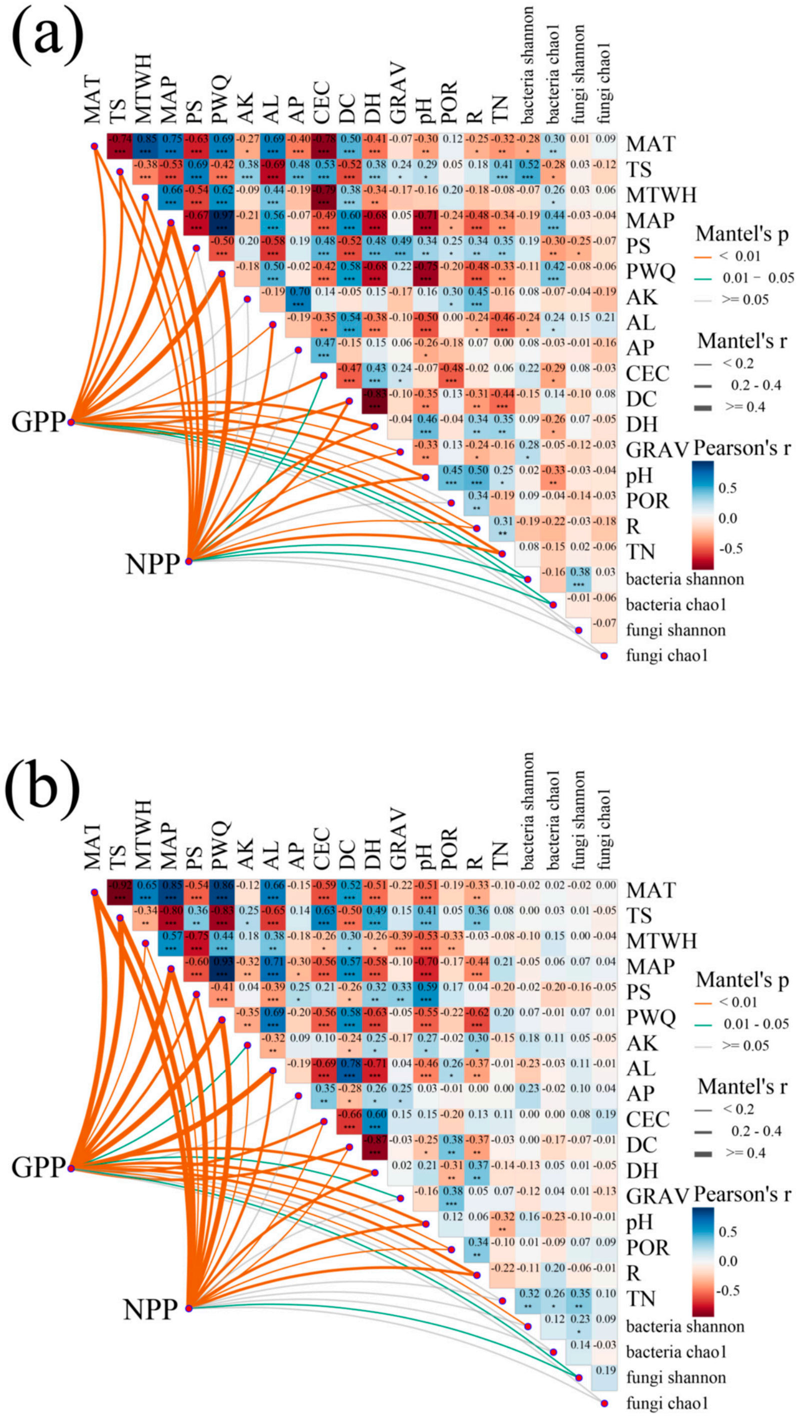

Figure. S2. Multivariate Correlation Analysis: Effects of Potential Influencing Factors on GPP and NPP in Natural Forests (a) and Plantation Forests (b). The influencing factors include:

Annual Mean Temperature (MAT), Annual Precipitation (MAP), Temperature Seasonality (TS), Precipitation of Wettest Quarter (PWQ), Max Temperature of Warmest Month (MTWM), Precipitation Seasonality (PS), Available K (AK), Exchangeable Al (AL), Available P (AP), Cation Exchange Capacity (CEC), Dry Color (DC), Soil Humidity (DH), Soil pH (pH), Soil Organic Carbon (SOC), Porosity (POR), Total Nitrogen (TN), Soil Particles (GRAV), Root Abundance (R), bacteria shannon, bacteria chao1, fungi shannon, fungi chao1. Asterisks indicate the significance level (***$p < 0.001$; **$p < 0.01$; *$p < 0.05$).

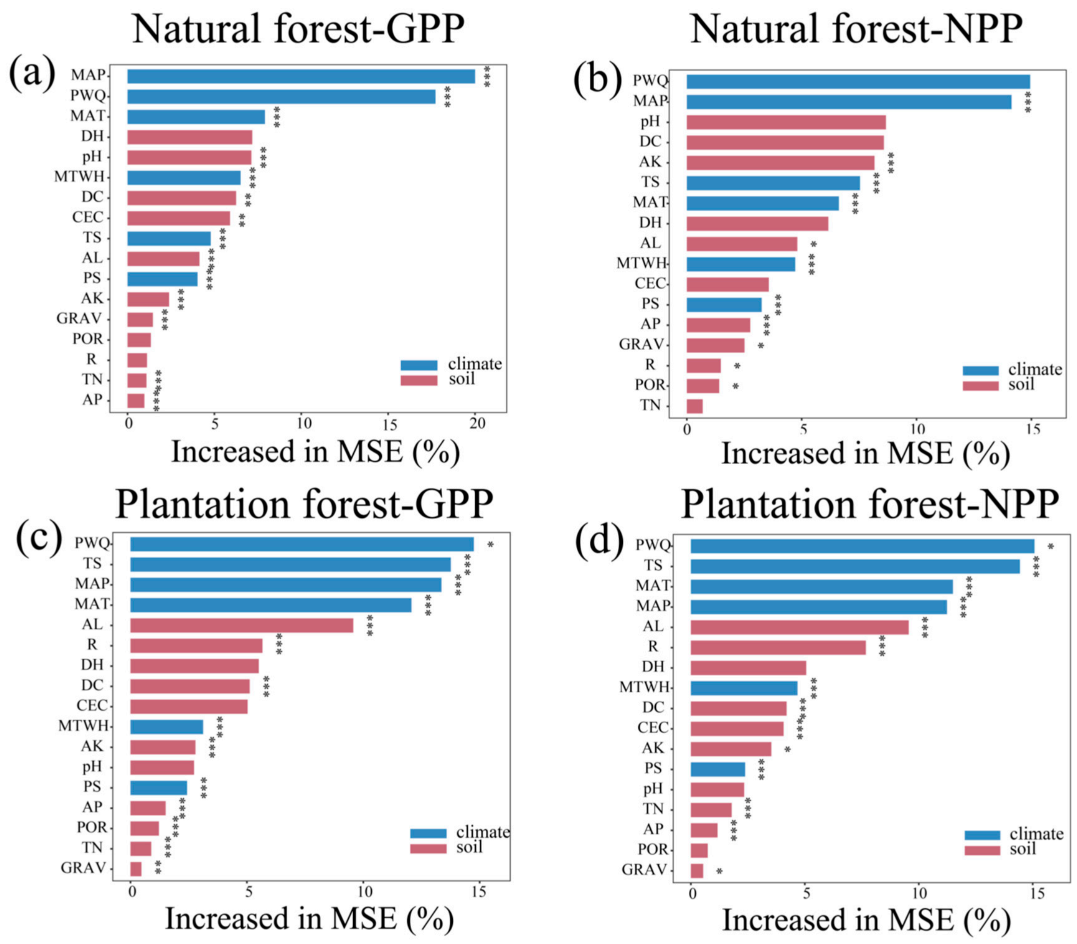

Figure. S3. Analysis of the Independent Contributions of Climatic Factors and Soil Nutrient Factors to Forest Productivity (GPP, NPP): (a) Natural Forests; (b) Plantation Forests. Climatic factors include: Annual Mean Temperature (MAT), Annual Precipitation (MAP), Temperature Seasonality (TS), Precipitation of Wettest Quarter (PWQ), Max Temperature of Warmest Month (MTWM), Precipitation Seasonality (PS); Soil nutrient factors: Available K (AK), Exchangeable Al (AL), Available P (AP), Cation Exchange Capacity (CEC), Dry Color (DC), Soil Humidity (DH), Soil pH (pH), Soil Organic Carbon (SOC), Porosity (POR), Total Nitrogen (TN), Soil Particles (GRAV), Root Abundance (R); Microbial diversity: bacteria shannon, bacteria chao1,

fungi shannon, fungi chao1. The importance of these variables was estimated by the percentage increase in the Mean Squared Error (MSE, %). Asterisks indicate the significance level (***$p < 0.001$; **$p < 0.01$; *$p < 0.05$).
